# First person – Rashmi Sivasengh

**DOI:** 10.1242/bio.062131

**Published:** 2025-07-18

## Abstract

First Person is a series of interviews with the first authors of a selection of papers published in Biology Open, helping researchers promote themselves alongside their papers. Rashmi Sivasengh is first author on ‘ Live-cell GLUT4 translocation assay reveals Per3 as a novel regulator of circadian insulin sensitivity in skeletal muscle cells’, published in BIO. Rashmi is a PhD student (final year) in the lab of Dr Brendan Gabriel at University of Aberdeen, Aberdeen, UK, investigating how cellular metabolism, circadian rhythms and lipid signalling interact in the context of metabolic disease.


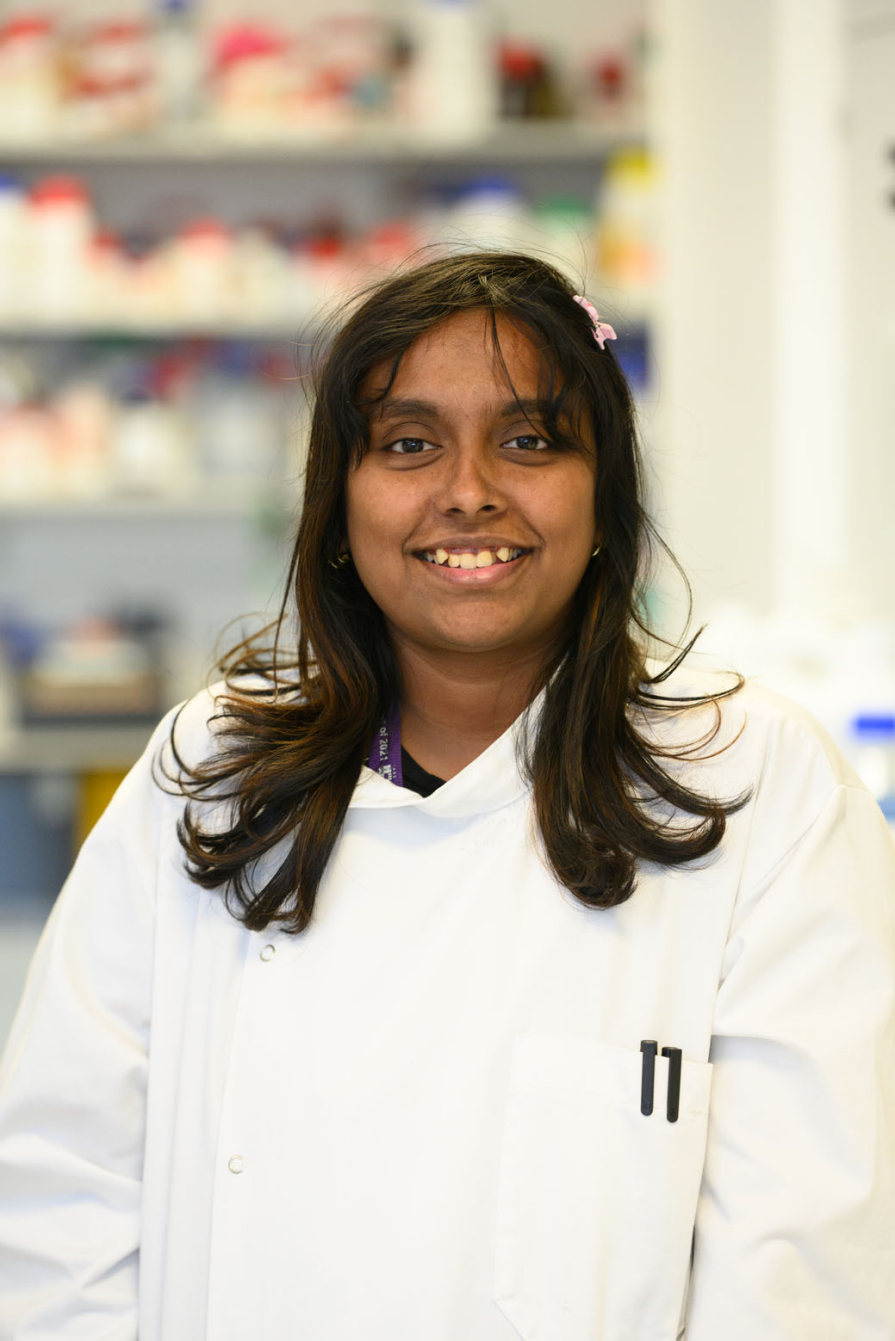


**Rashmi Sivasengh**

**Describe your scientific journey and your current research focus**

My interest in biomedical research began at the age of 10, when my close family member was diagnosed with type 2 diabetes and began long-term treatment with metformin. The prospect of improving lives through science filled me with purpose and drive, and it was at that point I decided to pursue biotechnology. During my undergraduate studies, I was deeply inspired by Dr Stephan Raj, a visiting professor from Stanford, who shared the story of DNA discovery through to the emerging field of RNA biology. This fuelled my curiosity in fundamental biology and the many unanswered questions that continue to challenge our understanding. I pursued a master's degree in industrial biotechnology to understand how discoveries move from bench to bedside and trained for 3 years in biopharmaceutical development at Biocon and Enzene Biosciences, focusing on biosimilar monoclonal antibody production. This industry experience sharpened my technical skills and reinforced the importance of bridging basic biology with translational outcomes. It was during this time that I discovered Dr Brendan Gabriel's lab, where research on exercise physiology and metabolic disease elegantly bridges fundamental biology with translational outcomes. The lab's focus on skeletal muscle biology, obesity and type 2 diabetes aligned perfectly with my growing interest in uncovering the mechanisms behind metabolic disorders.

**Who or what inspired you to become a scientist?**

I've always believed that health is the greatest wealth – a belief shaped by witnessing my father's lifelong journey with type 2 diabetes. His diagnosis and daily management with metformin from an early age inspired my curiosity in science and my desire to contribute to finding long-term solutions for chronic metabolic diseases. I often reflect on the impact of scientists like Frederick Banting and John Macleod, whose discovery of insulin over a century ago continues to save millions of lives. Their work reminds me that science has the power to outlive the scientist. It is this enduring legacy, of discovery that transcends time, that motivates me to become a scientist who can help improve lives, even when I am no longer around.

**How would you explain the main finding of your paper?**

Our bodies have an internal clock that helps regulate daily rhythms from sleep to metabolism. In people with chronic conditions like type 2 diabetes or obesity, this body clock often becomes disrupted. One key issue in diabetes is how the body moves sugar from the blood into cells. A protein called GLUT4 plays an important role in this process, but when it doesn't work properly, blood sugar stays high, leading to health problems. In our study, we developed a new method to track how GLUT4 moves inside live muscle cells over time. Using this, we discovered that GLUT4 movement also follows a daily rhythm and that certain genes may control this rhythm. These findings could help us better understand how the body clock affects sugar use in muscle, and how this might go wrong with diabetes.

**What are the potential implications of this finding for your field of research?**

Our live-cell assay provides a valuable tool for high-throughput screening to identify genes, pathways, or compounds that influence GLUT4 translocation, a key step in glucose uptake. Since skeletal muscle is responsible for nearly 80% of insulin-stimulated glucose disposal in the body, this platform has strong potential to advance our understanding of insulin resistance and metabolic disorders. It could also support the discovery of new therapeutic targets for conditions like type 2 diabetes by enabling functional validation of candidate regulators in a physiologically relevant cell model.

While it's well known that humans follow a sleep–wake cycle driven by an internal clock, it was truly fascinating to witness a similar rhythmic pattern in glucose uptake at the cellular level, outside the body

**Figure:**
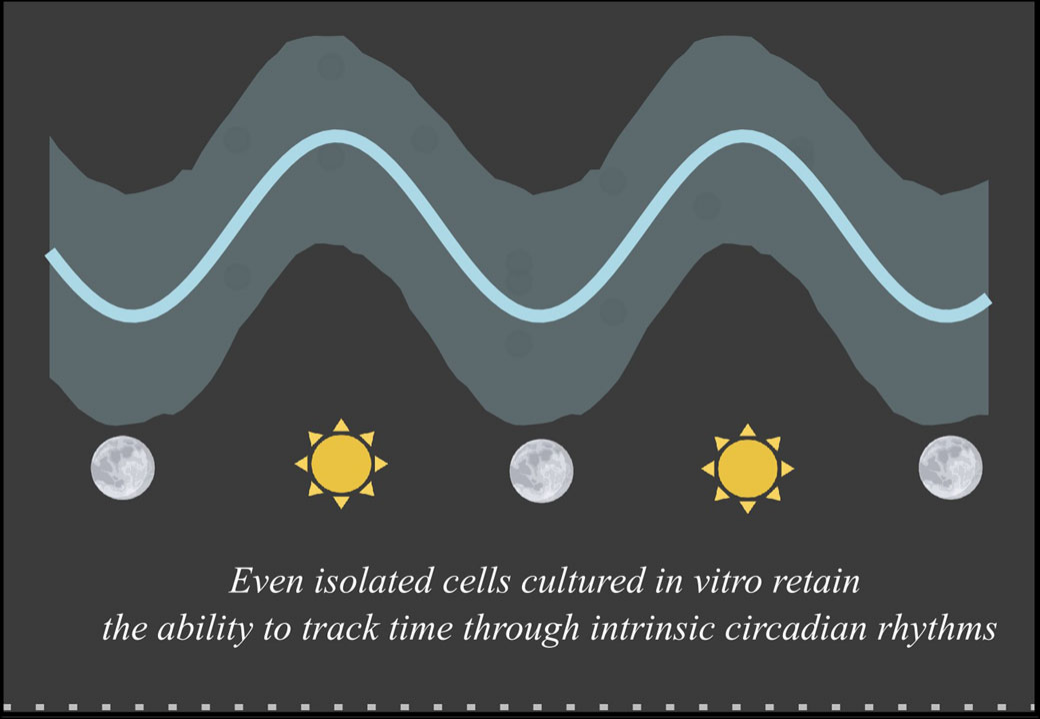
Remarkably, even single cells exhibit intrinsic circadian rhythms that mirror the sleep–wake cycle at the organismal level.

**Which part of this research project was the most rewarding?**

The most rewarding part of this project was observing a clear circadian rhythm in skeletal muscle cells cultured in a dish. While it's well known that humans follow a sleep–wake cycle driven by an internal clock, it was truly fascinating to witness a similar rhythmic pattern in glucose uptake at the cellular level, outside the body. Seeing live muscle cells display this time-of-day-dependent behaviour, especially in how they regulate glucose transporter activity, was both unexpected and exciting. It was a powerful reminder that even at the single-cell level, biological timekeeping plays a fundamental role.

**What do you enjoy most about being an early-career researcher?**

One of the most rewarding aspects of being an early-career researcher is the freedom to explore new ideas and try a wide range of experiments in the lab. I've found that this phase of research is not only rich with learning, but also filled with support and encouragement. Unlike more senior roles that often come with administrative responsibilities and less hands-on work, early-career researchers often receive the most appreciation and direct mentorship. I believe that freedom, combined with encouragement and guidance, creates the best environment to grow and achieve more.

Don't be afraid to fail, and don't let failure discourage you – it's an essential part of the research journey

**What piece of advice would you give to the next generation of researchers?**

Don't be afraid to fail, and don't let failure discourage you – it's an essential part of the research journey. In science, even a ‘failed’ experiment can provide valuable insight; negative results are still results, and sometimes they lay the foundation for the next big breakthrough. One piece of advice from my mentor that has stayed with me is this: “Whenever you face rejection – whether it's a paper or a grant – that's the moment to go back and read the success emails you've received”. It's a reminder to keep perspective and celebrate progress. Maintaining that balance is what helps sustain this rewarding path in research.

**What's next for you?**

I hope to continue doing impactful, curiosity-driven research in any platform or opportunity that comes my way. My long-term goal is to contribute to the development of pharmacological solutions for chronic metabolic conditions, such as diabetes, and ultimately help make life easier for people living with these disorders. Whether in academia or industry, I'm excited to be part of research that translates fundamental discoveries into real-world outcomes.

**Is there a personal reason that deepened your commitment to research?**

Yes. Recently, my partner was diagnosed with type 1 diabetes and is now undergoing insulin therapy. Witnessing the daily effort required to manage a chronic condition has profoundly reinforced my belief in the importance of scientific progress. It is only because of past research breakthroughs that managing such conditions is possible today. This experience has further strengthened my motivation to contribute to science – with the hope that future discoveries will not only improve treatment but ultimately bring us closer to a cure, making the world a better place to live.
